# Non-Invasive Monitoring of *Streptococcus pyogenes* Vaccine Efficacy Using Biophotonic Imaging

**DOI:** 10.1371/journal.pone.0082123

**Published:** 2013-11-20

**Authors:** Faraz M. Alam, Colin Bateman, Claire E. Turner, Siouxsie Wiles, Shiranee Sriskandan

**Affiliations:** 1 Infectious Diseases and Immunity, Department of Medicine, Imperial College London, London, United Kingdom; 2 Department of Molecular Medicine and Pathology, University of Auckland, Auckland, New Zealand; Universidad Nacional de La Plata., Argentina

## Abstract

*Streptococcus pyogenes* infection of the nasopharynx represents a key step in the pathogenic cycle of this organism and a major focus for vaccine development, requiring robust models to facilitate the screening of potentially protective antigens. One antigen that may be an important target for vaccination is the chemokine protease, SpyCEP, which is cell surface-associated and plays a role in pathogenesis. Biophotonic imaging (BPI) can non-invasively characterize the spatial location and abundance of bioluminescent bacteria *in vivo*. We have developed a bioluminescent derivative of a pharyngeal *S. pyogenes* strain by transformation of an *emm*75 clinical isolate with the *luxABCDE* operon. Evaluation of isogenic recombinant strains *in vitro* and *in vivo* confirmed that bioluminescence conferred a growth deficit that manifests as a fitness cost during infection. Notwithstanding this, bioluminescence expression permitted non-invasive longitudinal quantitation of *S. pyogenes* within the murine nasopharynx albeit with a detection limit corresponding to approximately 10^5^ bacterial colony forming units (CFU) in this region. Vaccination of mice with heat killed streptococci, or with SpyCEP led to a specific IgG response in the serum. BPI demonstrated that both vaccine candidates reduced *S. pyogenes* bioluminescence emission over the course of nasopharyngeal infection. The work suggests the potential for BPI to be used in the non-invasive longitudinal evaluation of potential *S. pyogenes* vaccines.

## Introduction

*Streptococcus pyogenes* is estimated to be responsible for over 600 million new cases of pharyngitis each year [1]. In addition to triggering autoimmune sequelae, such as acute rheumatic fever, nasopharyngeal infection with *S. pyogenes* represents the major reservoir for invasive diseases such as necrotizing fasciitis, pneumonia and toxic shock syndrome that together cause an estimated 163,000 deaths worldwide each year [1]. 

A number of vaccine candidates are currently in development for *S. pyogenes*, although none have yet been licensed [2]. Many of these are based upon the surface expressed M-protein [3,4], although antigenic variability is an obstacle to development [5] coupled with fears regarding immunological cross-reactivity with human proteins. Some vaccines focus on more broadly conserved antigens, such as C5a peptidase [6], fibronectin binding proteins [7,8], or a combination of bacterial components [9,10].

SpyCEP is known to play a key role in invasive infection, enabling *S. pyogenes* to spread systemically [11]. The mitigation of its effects by vaccination can benefit host survival after invasive intravenous, intramuscular and lower respiratory tract infection with *S. pyogenes* [9,10,12,13]. It is known that SpyCEP is surface expressed, although the potential of a SpyCEP-based vaccine to increase bacterial clearance and confer protection against infection in the nasopharynx has not yet been evaluated longitudinally.

Biophotonic imaging (BPI) [14] enables the non-invasive temporal quantification and spatial localization of bioluminescent bacteria as an infection develops. The application of longitudinal BPI to such infections can refine *in vivo* modelling, and reduce the numbers of animals required for research [15]. BPI has been used to study the impact of immunization on longitudinal infection with an *emm*49 isolate of *S. pyogenes* [16] in the context of nasal [6] and peritoneal infection [4].

We have previously developed a model of nasopharyngeal infection using a clinical *emm*75 *S. pyogenes* pharyngitis isolate [17]. Here, we describe the development and evaluation of a bioluminescent derivative of this clinical *S. pyogenes* isolate and the application of BPI to determine the impact of vaccination on infection of the nasopharynx by *S. pyogenes*. 

## Methods

### Ethics statement

*In vivo* experiments were performed in accordance with the Animals (Scientific Procedures) Act 1986, and were approved by the Imperial College Ethical Review Process (ERP) panel and the UK Home Office.

### Bacterial strains

Isogenic derivatives of an *S. pyogenes emm*75 isolate (H347) were used in all studies. *S. pyogenes* was cultured on Columbia Blood Agar (CBA), Todd Hewitt agar (THA), Todd Hewitt Yeast (THY) broth or C-medium [18]. *E. coli* strains DH5α and BL21, used to propagate plasmids and express protein, were cultured in Luria Bertani (LB) broth. 

### Plasmids and construction of bioluminescent *S. pyogenes* strains

#### pICL18^lux^

**Figure 1:**
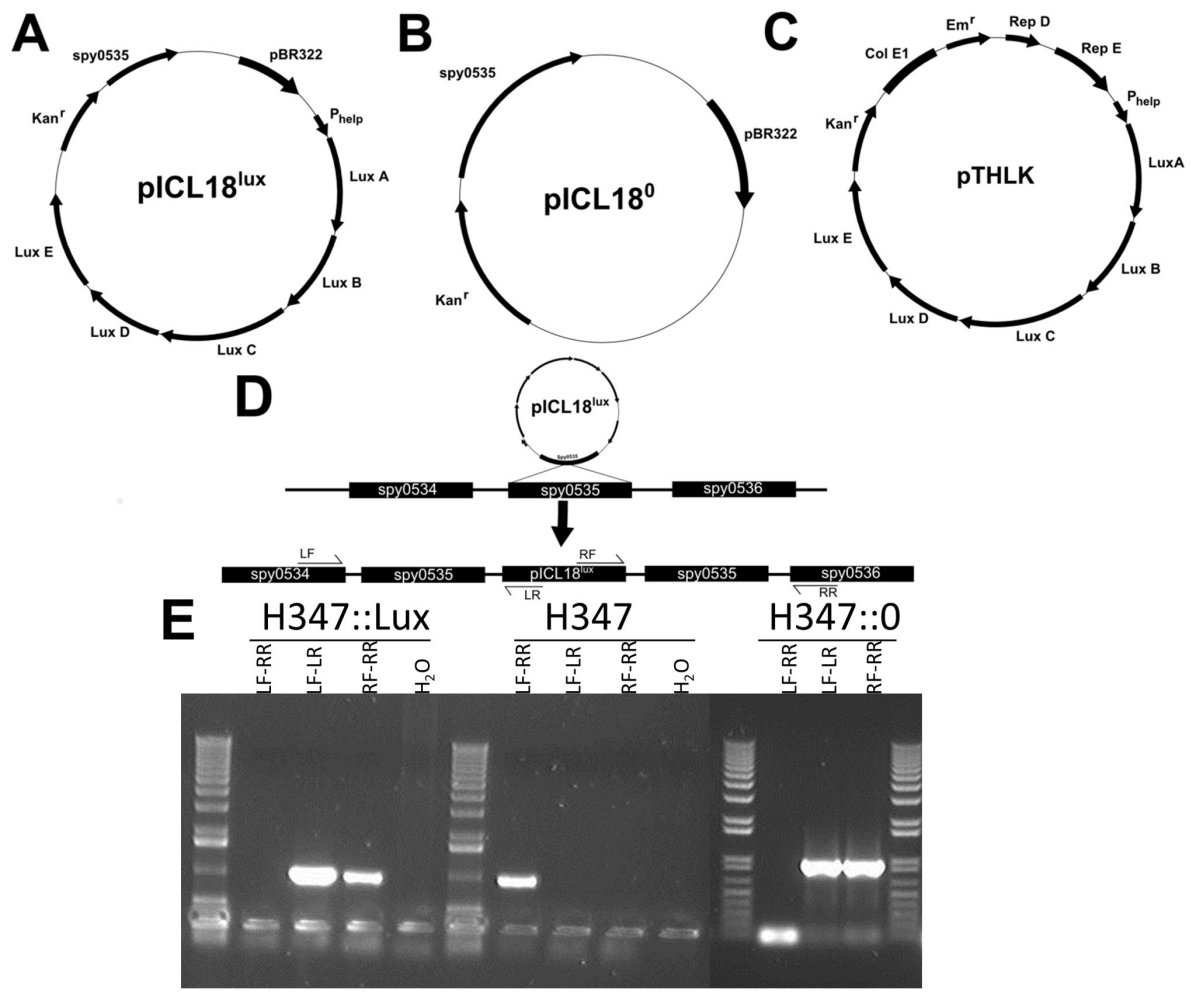
Plasmids and integration of pICL18lux into the S. *pyogenes* chromosome. The integrating plasmids pICL18^lux^ (A) and pICL18^0^ (B) and the replicating plasmid construct pTHLK (C) are demonstrated, with arrows indicating locations and orientations of open reading frames. A diagrammatic representation of the integration of the plasmid pICL18^lux^ into the S. *pyogenes* chromosome via a single crossover is shown with the targets for the diagnostic primers LF, LR, RF and RR. Integration will produce positive results for the regions between LF-LR and RF-RR (D). PCR using these primers was performed on DNA purified from H347::lux, H347 and the H347::0 (E) and confirmed integration of pICL18^lux^ and pICL18^0^ into the S. *pyogenes* genome at the Spy0535 locus.

The *luxABCDE* operon was amplified from the plasmid pSB2025 [19] using primers for the leading sequence of *luxA* (CGCGCCCGGGCCATGGGCAGTCGACAGGAGTCTCTATGAAATTG) and the closing sequence of *luxE* (TTAGAGCTCGCGGCCGCGATATCAACTATCAAACGC). These were ligated into pUC18N [20] through restriction digestion with Nco1/Pac1 enzymes (New England Biosciences) creating pUC18N+*luxABCDE*. The P_help_ promoter sequence [21] was synthesized by DNA 2.0 in the vector pJ201:19300 and amplified using primers P_help_ F (CGATCTTAGGATCCCCCGGGG) and P_help_ R (TGTAGGTTGGTACCGCGGCCG) then ligated upstream of *luxA* in tpUC18N+*luxABCDE* using NcoI/PacI restriction digestion. The *apha3* kanamycin resistance gene was amplified from pUCMUT [22] using *apha3 F* (TTAATTAAAGCGAACCATTTGAGGTGAT) and *apha3 R* (GAGCTCTGGACAGTTGCGGATGTACT) and ligated into this vector via a PacI/SacI restriction digest. The conserved *S. pyogenes spy0535* gene (accession no. NP_268809) identified by Park et al [16] as a target of insertion was amplified from *S. pyogenes* strain H347 DNA using the Spy F primer (GAGCTCCAATTAAAAGTTGTGAATTACGAATGA) and Spy R primer (GGTACCTCCTCACTACTTCTGCCTTTTTG). This DNA fragment was ligated into the plasmid described above via a Sac1/Kpn1 restriction digest. The *bla* ampicillin gene resistance was excised through restriction digestion with PvuI, to create pICL18^lux^ (Figure 1A). Loss of the *bla* ampicillin resistance gene was confirmed by susceptibility to ampicillin following successive streaking of pICL18lux-transformed *E. coli* onto selective media. 

#### pICL18^0^

To control for insertion of the plasmid into the *spy0535* region, the *luxABCDE* operon was excised from pICL18^lux^ via an EcoRI/BglII restriction digest. Linker primer F (AATTCGGCGGGACTAGTCGGGGA) and Linker primer R (GATCTCCCCGACTAGTCCCGCCG) were mixed in equal molar concentrations within molecular grade water and heated to 90°C before being cooled to room temperature to produce an oligonucleotide linker. This linker was inserted into the plasmid to create pICL18^0^ (Figure 1B). 

#### pTHLK

To construct a plasmid for multi-copy expression of the *lux* operon, plasmid pIB184 [23] was first digested with ApaI and BamHI, blunted (NEB Blunting Kit) and self-ligated to remove the P_23_ promoter. A linear construct comprising the P_help_ promoted *luxABCDE* operon and *aph3a* gene was obtained from pICL18^lux^ and inserted into the unpromoted pIB184 derivative via EcoRI/SacI digestion and ligation to create the replicating plasmid pTHLK (Figure 1C).

*S. pyogenes* was transformed with plasmids pICL18^lux^, pICL18^0^ and pTHLK by electroporation as described previously [24] to create the strains H347::lux, H347::0 and H347pTHLK, respectively. Transformants were selected on agar containing kanamycin, and screened using bioluminescence where appropriate. Integration of the pICL18 derived plasmids into the S. *pyogenes* chromosome via a single crossover event (Figure 1D) was confirmed using diagnostic primers LF (CAAAAGATGATGTTGTCCTAATTCA) LR (GTTTTCCCAGTCACGACGTT), RF (CCGCAACTGTCCATACTCTG), and RR (GCTTTTCTATACTATCCCCTTCTTTC) shown in Figure 1 E.

### Characterization of bioluminescent strains

Overnight cultures of *S. pyogenes* strains H347::lux and H347pTHLK were re-suspended in phosphate buffered saline (PBS), and tenfold serial dilutions used to quantify bioluminescence intensity (given as photons s^-1^) using the IVIS100 system (PerkinElmer). Bacterial numbers were also assessed via retrospective plating onto selective agar.

Growth rates of each strain were assessed in THY broth (n=6 per strain). Bacterial growth was quantified at hourly intervals by measuring optical density at 600 nm (OD_600_) (Biowave, CO 8000 Cell density meter). Luminescence (given as Relative Light Units [RLU]) was quantified using a Modulus Single Tube Luminometer (Turner Biosystems).

### Stability of bioluminescence expression

The stability of the replicative and integrated plasmids in *S. pyogenes* was assessed by serial passage in liquid media. An inoculum prepared from overnight cultures containing 10^7^ colony forming units (CFU) of either H347pTHLK or H347::lux was inoculated into THY broth without antibiotic selection (n=3). Every 24 hours, 1 ml of each culture was inoculated into 49 mls of fresh THY broth with the same growth conditions as the previous culture. Identical aliquots were taken at regular intervals over the course of 7 days to quantify the bacteria that had retained the constructs at each time point, by plating into THA with and without antibiotic selection.

### Animals

Female 4-5 week old FVB/n mice (Harlan, UK) were maintained in individually HEPA filtered cages with sterile bedding and free access to sterilized food and water. GLP Mini Fun Tunnels (Lillico) were provided in each cage for environmental enrichment. Mice were weighed daily; reduction by 20% of original weight was a defined humane endpoint.

### *In vivo* biophotonic imaging

Bioluminescence from infected animals was assessed under isoflurane anaesthesia using the Xenogen IVIS 100 camera system (Perkin Elmer). For anatomical localization, a pseudocolour image representing light intensity (blue, least intense to red, most intense) was generated using the Living Image software and superimposed over the grayscale reference image (displayed as photons s^-1^ cm^-2^ sr^-1^). Images were gated at a Radiance of 4000 photons s^-1^ cm^-2^ steradian (sr)^-1^ to eliminate potential background sources of bioluminescence. Total Flux (as photons s^-1^) within specific regions of individual mice was also quantified using the region of interest (ROI) tool in the Living Image software program (Version 2.0, Perkin Elmer). 

### Intranasal inoculation

An inoculum of 10^7^ CFU in a 5μl volume was administered to mice under isoflurane anaesthesia via the intranasal route using a pipette (2.5μl per nostril). Numbers of viable bacteria within the inoculum were retrospectively assessed by plating onto CBA. The nasopharynx was homogenized into PBS and plated onto CBA to quantify streptococci at the end of each experiment.

In some experiments, to monitor the intensity of nasopharyngeal infection longitudinally alongside imaging, nasal shedding onto CBA was monitored, as a surrogate marker of intensity of nasopharyngeal infection. Briefly, the anterior nares were gently applied to CBA plates 10 times and spread, then plates were incubated overnight and colonies of bioluminescent *S. pyogenes* were counted, as previously described [17]. The presence of *S. pyogenes* was confirmed by Gram staining, catalase testing and Lancefield grouping and, in the case of bioluminescent strains, by luminometry. 

In experiments to determine the relation between bioluminescent signal and bacterial burden in the nasopharynx, cohorts of mice were infected, then imaged on day 1, 2, or 3 post infection (n=6 per time point, performed 3 times, n=54). At each time point mice were euthanised and dissected to obtain nasopharyngeal tissue [17]. This tissue was then homogenised, plated onto CBA and cultured overnight to determine the total burden of *S. pyogenes*.  In a subset of 18 mice (n= 6 per time point, n= 18 total), dissected nasopharyngeal tissue and nasal associated lymphoid tissue (NALT) were imaged immediately following dissection, and then cultured on CBA separately to determine burden of *S. pyogenes* in each anatomical location in relation to bioluminescence.

### *In vivo* competition assay

H347, H347::lux or H347::0 were cultured overnight, centrifuged (1864×g for 10 min) and washed twice in PBS. The OD_600_ for each strain was adjusted to 100 (7×10^9^ CFU ml^-1^), and suspensions of different isolates were mixed 1:1 pairwise and used to inoculate groups of 8 mice. The mixtures were H347/H347::lux, H347/H347::0 and H347::Lux/H347::0. Bacterial colonies from the nasopharynx were replica plated onto selective media on day 1 (4 mice per group) or day 3 (4 mice per group). Individual colonies were inoculated into THY and bioluminescence quantified after 5 hours of growth (Modulus Single Tube Luminometer). *S. pyogenes* that were either bioluminescent or kanamycin resistant were quantified to determine the proportion of each strain surviving *in vivo*. Competitive Indices (CI) were calculated as follows: CI=(mutant output/WT output)/(mutant input/WT input).

### Vaccination protocol

Mice were immunized intramuscularly with 50μl of vaccine, comprising either heat-inactivated *S. pyogenes* strain H347 or SpyCEP protein, 4, 2 and 1 weeks prior to challenge. To prepare a heat-killed vaccine, *S. pyogenes* was heated for 1 hour at 80°C and concentrated to a dose of 10^8^ CFU per mouse. Sterility was verified by plating onto CBA.

Recombinant CEP (2mg ml^-1^) was mixed 1:1 with adjuvant to create the SpyCEP based vaccine [11]. As controls, groups of mice were immunized either with PBS alone, or with PBS mixed 1:1 with adjuvant. For priming vaccinations, Complete Freund's adjuvant was used, and subsequent boosters used Incomplete Freund's adjuvant, in order to replicate adjuvants used in a previous vaccination study [12].

### Measurement of antibody responses in serum

Murine IgG antibody responses were measured using ELISA based assays. Plates were coated with 10^8^ CFU ml^-1^ of heat-killed *S. pyogenes*, or 20μg ml^-1^ of recombinant CEP and bound IgG selective against these antigens was detected using HRP-conjugated goat anti-mouse IgG.

### Statistics

Regression analysis was used to fit data to the appropriate linear or logistic models to determine rate constants and to allow curve comparison. An r^2^>0.95 was determined to be a good fit for the data. Differences in bioluminescent signals between different groups *in vivo* over time were analysed by Repeated Measures Two Way ANOVA with Bonferroni correction. Mann-Whitney analysis was used to analyse differences in colony numbers between two groups. To analyse multiple groups, the Kruskal-Wallis test was used. *P* values less than 0.05 were determined to be significant. All analyses were performed using Graphpad Prism (5.0).

## Results

### Bioluminescence expression by *S. pyogenes in vitro*

**Figure 2:**
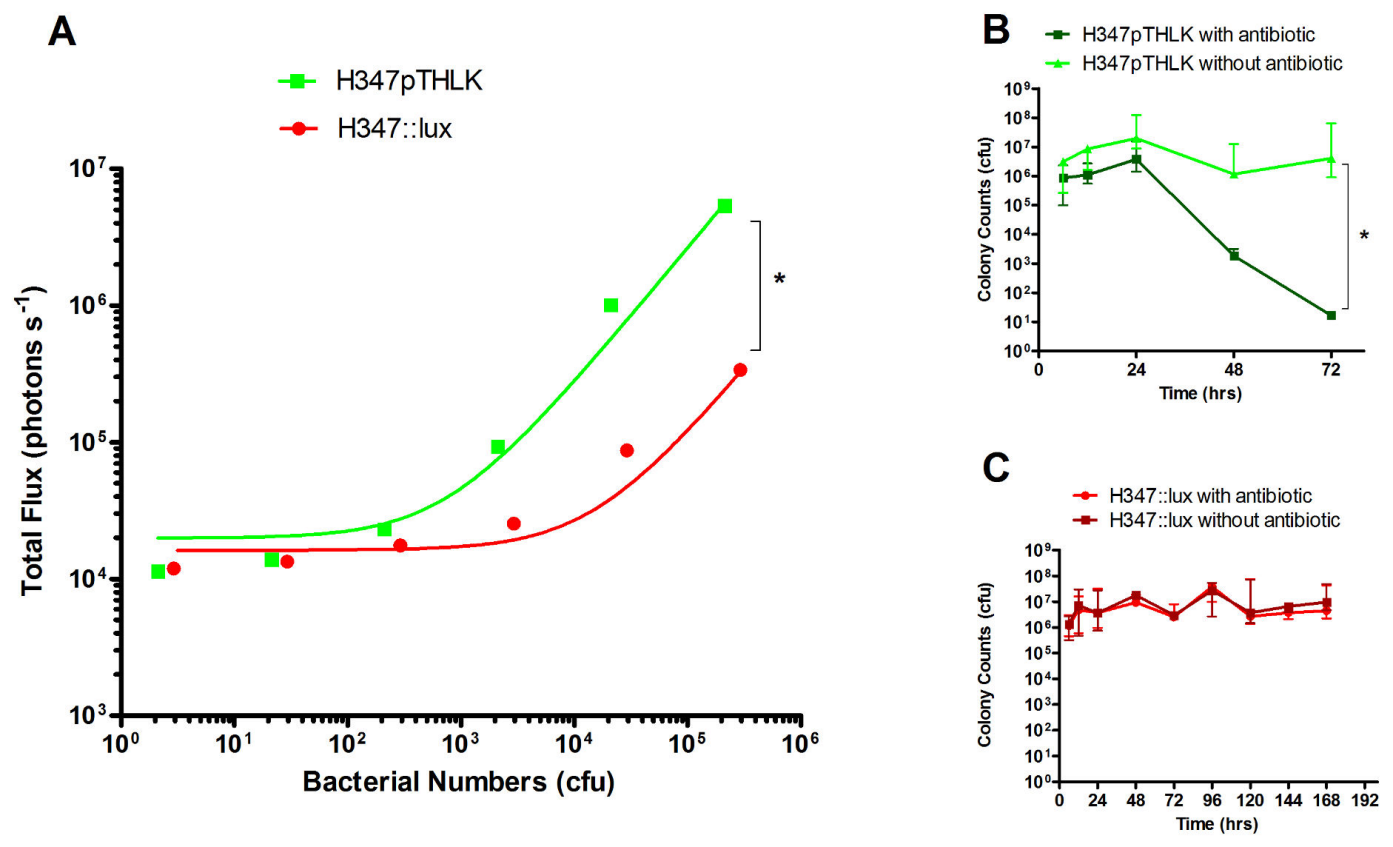
Characterization of bioluminescent strains *in* *vitro*. Serial dilutions of bioluminescent strains transformed with either plasmid (H347pTHLK) or integrated construct (H347::lux) were imaged in a 96 well plate (n= 3 separate cultures, Linear regression, r^2^>0.95, * *p* <0.05) using an IVIS 100 system (Perkin Elmer) to relate luminescence to bacterial abundance. Bacteria were quantified after plating onto agar (A). H347pTHLK (B) and H347::lux (C) were serially passaged *in* *vitro* without antibiotic over 7 days; Samples of each culture were plated onto kanamycin solid medium or onto non-antibiotic media and demonstrated a failure of H347pTHLK to grow when cultured on antibiotic containing agar after 72h, consistent with loss of the plasmid. n=3 separate cultures, Repeated measures Two way ANOVA with Bonferroni correction, * *p* <0.05). Error Bars indicate median and range.

*S. pyogenes* transformed with either the replicative plasmid pTHLK (H347pTHLK) or with the integrated pICL18^lux^ construct (H347::lux) were cultured to logarithmic phase and luminescence measured. There was a significant difference in light production between the two bioluminescent strains (Linear regression *p*<0.05.). H347pTHLK produced on average 26.19 (± 0.06 SE) photons s^-1^ CFU^-1^ whereas H347::lux produced on average 1.062 (± 0.012 SE) photons s^-1^ CFU^-1^ (Figure 2 A). 

During passage *in vitro*, the numbers of *S. pyogenes* carrying the pTHLK plasmid (H347pTHLK) declined a thousand-fold after each passage without antibiotic selection (Figure 2 B, Repeated measures Two way ANOVA with Bonferroni correction). In contrast, H347::lux retained the *luxABCDE* construct over a seven day period (Figure 2 C), without the presence of antibiotics. The stability of the integrated *luxABCDE* construct made it suitable for further study.

### The expression of bioluminescence impairs growth

The growth characteristics of H347::lux were compared to both an isogenic strain, H347::0, constructed through transformation with pICL18^0^, which integrates into the S. *pyogenes* genome at the *spy0535* site but does not confer bioluminescence, and the parental strain H347. 

**Figure 3:**
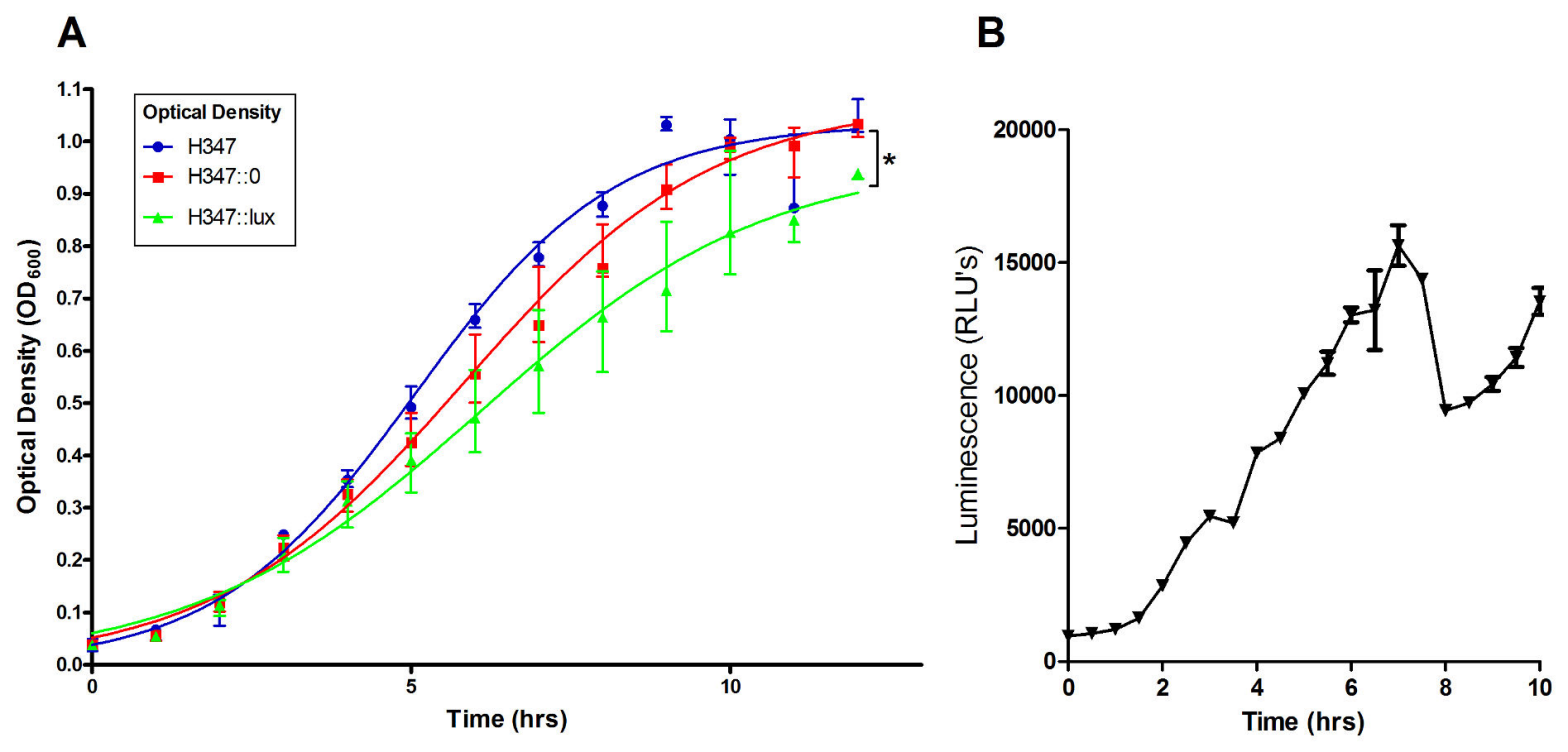
The effect of bioluminescence expression on *in* *vitro* growth. *S. pyogenes* strain H347::lux was cultured over a 10 hour period alongside the isogenic strains H347 and H347::0. Logistic curves were fitted to the data (A, n=6 per group, from experiments performed on two separate days, Linear Regression r^2^>0.95, * *p* <0.05). Luminescence of H347::lux was quantified during growth (B) Error bars indicate median and interquartile range.

Logistic regression analysis showed no significant difference in growth rate between the isogenic strains, but H347::lux demonstrated a significant reduction in the maximum growth capacity compared with the other strains (Figure 3 A p<0.05). During growth *in vitro*, bioluminescence from H347::lux peaked at 7 h post inoculation (Figure 3 B) and then declined steeply. This decrease has been previously reported for *luxABCDE*-expressing bacteria and reflects a reduction in bacterial metabolic activity due to the reduced availability of flavin mononucleotide that is required by the luciferase [25]. 

The expression of bioluminescence *in vivo* confers a competitive disadvantage in the nasopharynx.

**Figure 4:**
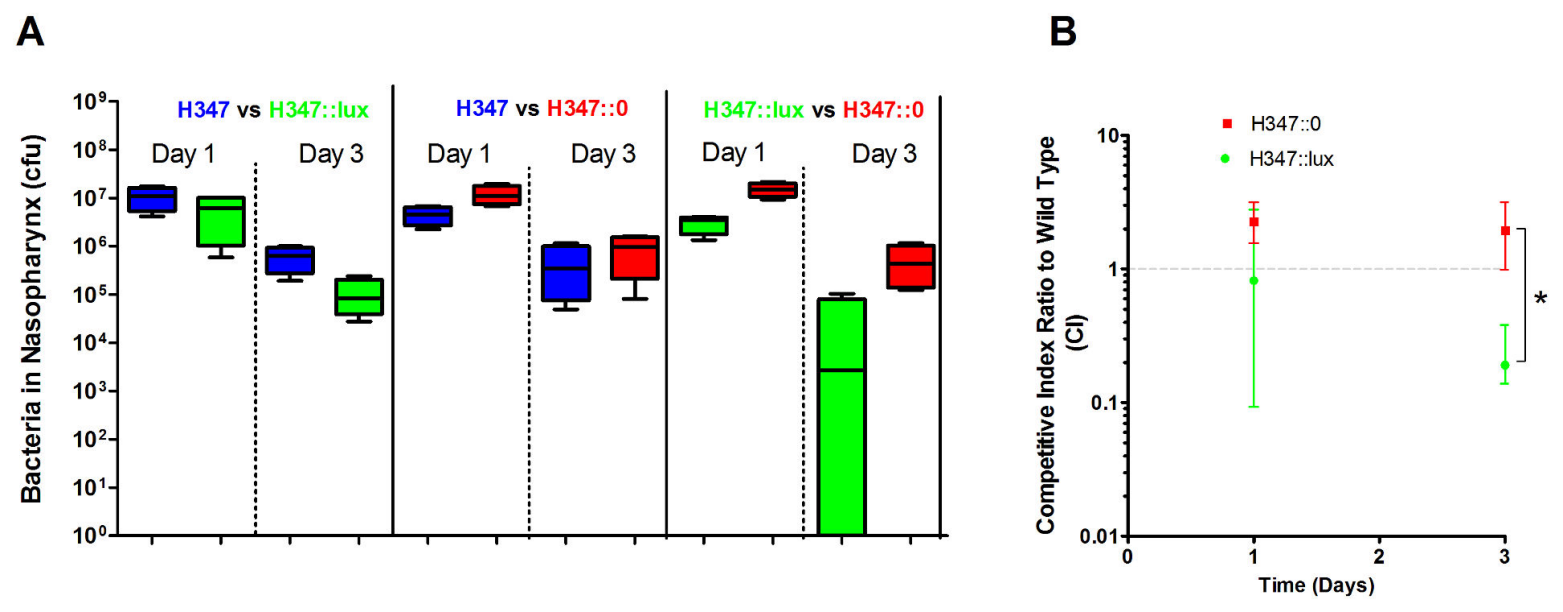
Bioluminescence confers *in* *vivo* competitive growth defects. Mice were infected intranasally using mixed inoculation paired combinations of H347::lux, H347, and H347::0 as shown. Mice were culled either at day 1 or day 3 post inoculation and the relative abundance of each strain was assessed through replica plating onto selective media (A, n=4 per group). These results were used to calculate competitive indices between each mutant strain and the isogenic parent strain H347 (B, Repeated measures Two way ANOVA with Bonferroni correction * *p*< 0.05). Error bars indicate median and interquartile range.

Inocula containing pairwise mixes of H347, H347::lux and H347::0 were administered intranasally (Figure 4 A, n=4 per group) to determine competitive survival *in vivo* at day 1 and day 3 post inoculation. The competitive index relative to the parental strain was calculated for H347::lux and H347::0 strains in the nasopharynx (Figure 4 B, Repeated measures Two Way ANOVA with Bonferroni correction). This showed that H347::lux was significantly attenuated over the time course compared to H347::0.

### Longitudinal monitoring of intranasal infection with bioluminescent *Streptococcus pyogenes*

**Figure 5:**
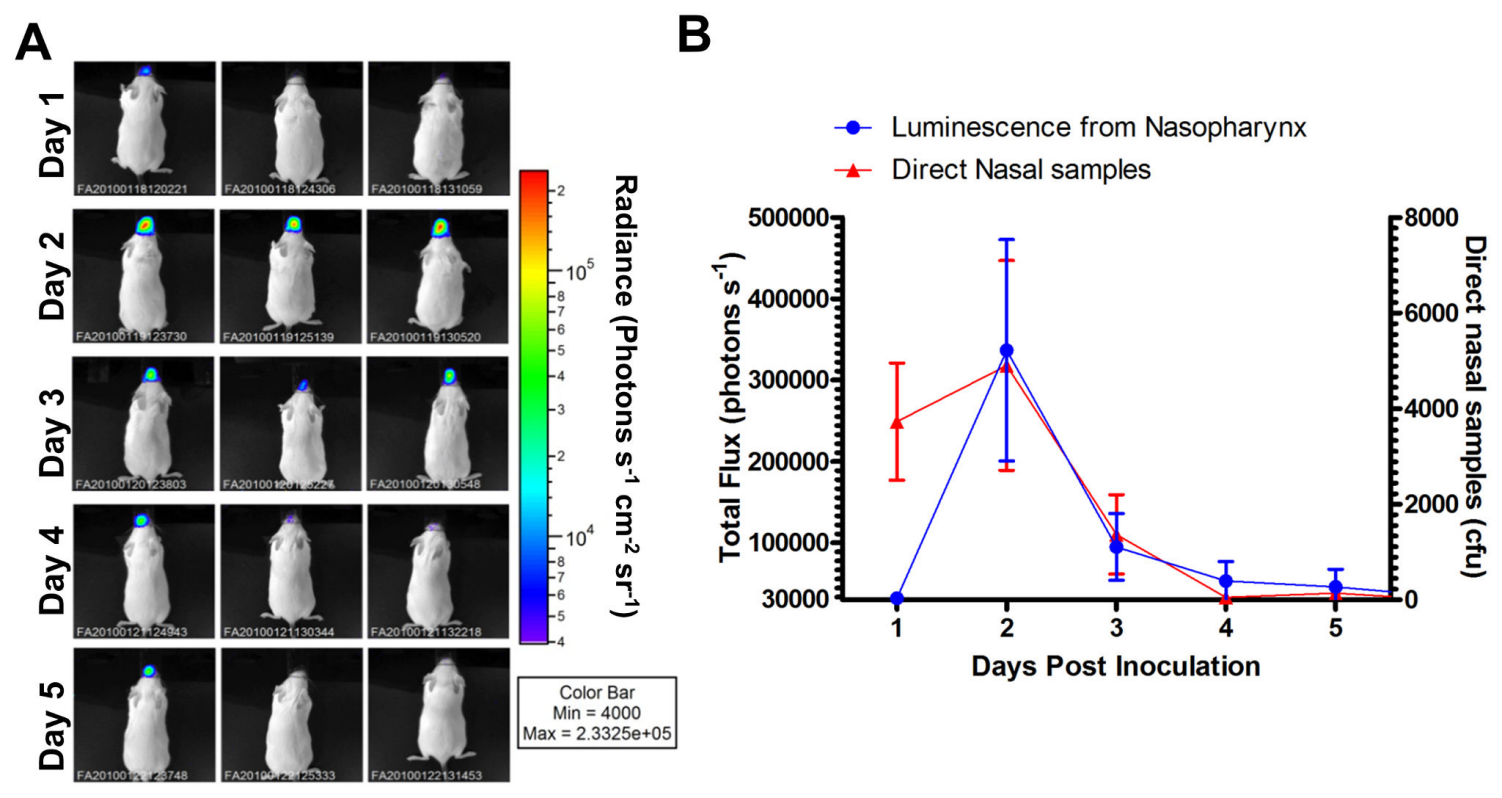
Imaging of *S. pyogenes* with a stably integrated lux operon over a time course. Mice (n=6) intranasally inoculated with *S. pyogenes* (H347::lux) were imaged over five days (A, three representative mice shown for each time point) to demonstrate anatomical localisation. Total flux from the nasopharynx was measured daily alongside longitudinal measurement of *S. pyogenes* nasal shedding (B, n=6).

Intranasal infection caused by H347::lux was studied over the course of five days using six mice (Figure 5 A). The luminescent signal dissipated to background levels in the majority of mice after 4 days of infection, alongside a drop in bacterial colonies recovered from direct nasal samples (Figure 5 B). Dissections revealed an average of 64228 (±38491 SEM) CFU of *S. pyogenes* within the nasopharynx at day 5 post infection.

### The relationship of bioluminescence to bacterial burden within the nasopharynx

**Figure 6:**
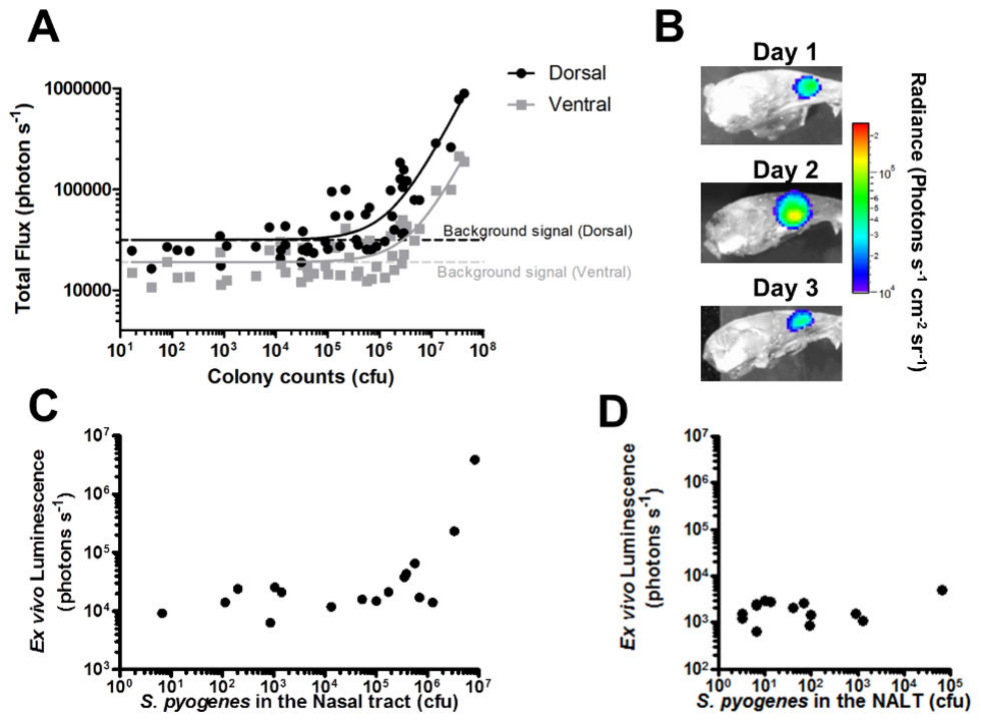
Relationship of bioluminescent signal to *S. pyogenes* colony counts. Dorsal and ventral luminescence measurements obtained *in* *vivo* during a three day intranasal infection were compared to bacterial numbers obtained on dissection at one, two and three days post infection. (A, Linear Regression, r^2^> 0.95, n=54 from three separate experiments performed on different weeks). The nasopharynx was imaged, following dissection, to demonstrate anatomical localisation of the BPI signal.to the posterior turbinates (B). Bioluminescence of dissected nasal tissue (C) and NALT (D) was plotted against actual *S. pyogenes* burden obtained from these tissues following dissection and culture. Bioluminescent signal from dissected NALT was plotted against the numbers of *S. pyogenes* within nasal tissue and NALT (n=18 each group. Linear Regression, r^2^ >0.95 for nasal tissues and, r^2^ <0.95 for NALT). Data points shown for individual mice.

To determine if bioluminescence expressed in the nasopharynx would correlate with bacterial burden, intranasally infected mice were culled at days 1, 2 and 3 post inoculation. Light intensity detected from the dorsal and ventral viewpoints of the mouse nasopharynx correlated to *S. pyogenes* abundance upon dissection (Figure 6 A, n=54, r^2^ > 0.95), demonstrating that bioluminescence can be used to quantitatively assess carriage. The data revealed a background signal of 31701 ± 6731 photons s^-1^ emanating from the nasopharynx. In practice, this generates a limit of detection of approximately 5 ×10^5^ CFU when imaging mice from the dorsal viewpoint.

BPI also revealed the ethmoturbinates to be the primary anatomical location of *S. pyogenes* during the first 3 days of nasal infection (Figure 6 B), and *S. pyogenes* numbers within the ethmoturbinate tissue linearly correlated with the signal obtained from mice *ex vivo* (Figure 6 C, n=18, r^2^ >0.95). The signal obtained from the nasal associated lymphoid tissue (NALT) did not correlate to the detected luminescence (Figure 6 D, n=18) possibly because the burden was too low to be detected. 

### Evaluation of heat inactivated *S. pyogenes* vaccine in a nasal challenge model using BPI

**Figure 7:**
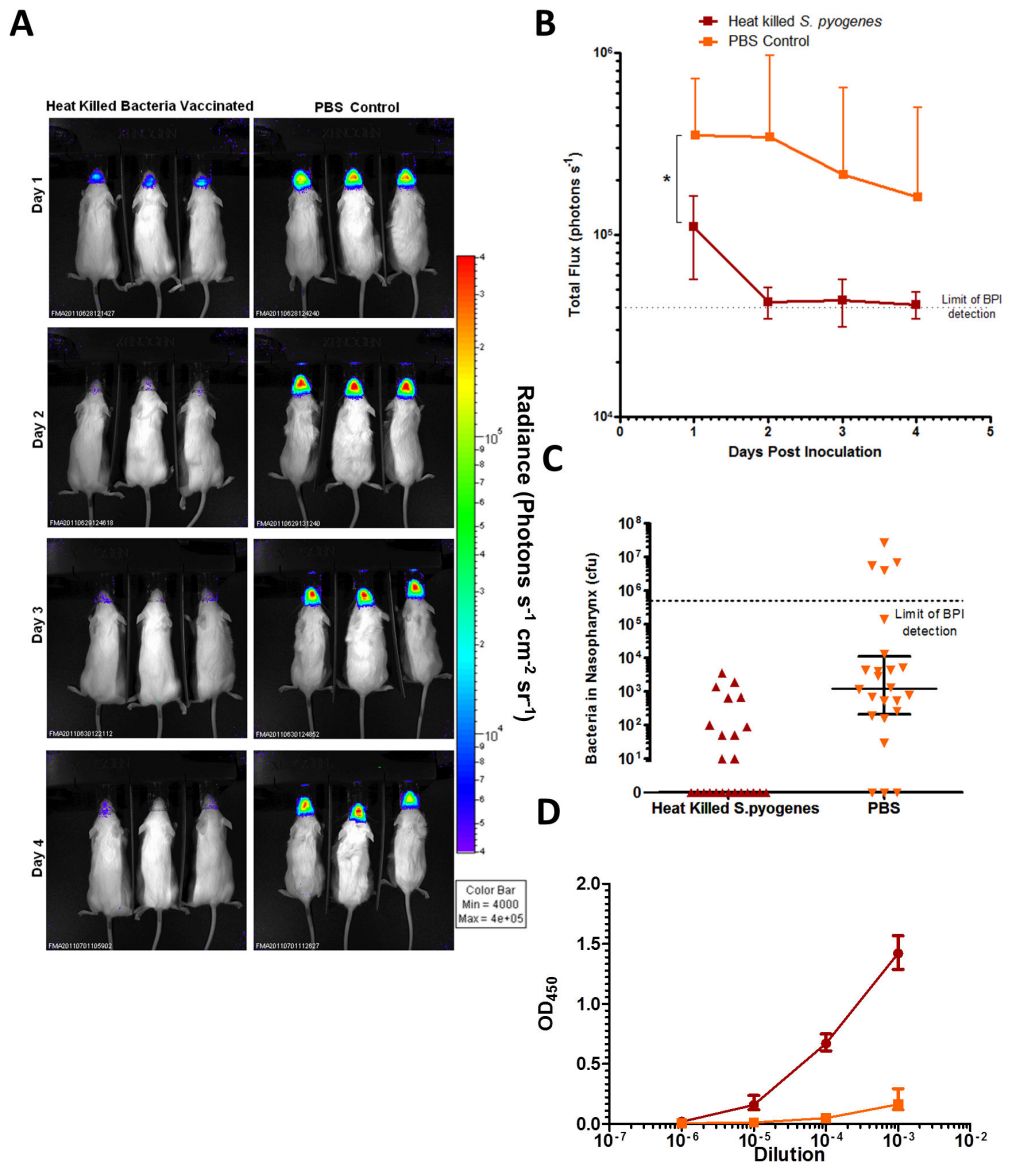
BPI to confirm the protective efficacy of a vaccine based on heat-killed bacteria. Mice were immunized with either PBS or with heat-killed *S. pyogenes* strain H347, and then challenged with intranasal H347::lux. The mice were imaged for 4 days post infection using an IVIS 100 system (A). Total flux from mice in each group was compared over the time course (B, Repeated Measures Two way ANOVA with Bonferroni post-test * *p*<0.05). Colony counts were obtained from dissections performed on the final day of infection (C, Mann-Whitney, * *p*<0.05). Serum antibody responses against *S. pyogenes* from tail bleeds taken before infection were measured by ELISA (D). Error bars indicate mean and SD.

Following vaccination with heat-inactivated *S. pyogenes*, mice were challenged intranasally with H347::lux and imaged for 4 days post inoculation (Figure 7 A).

Mice immunized with heat-inactivated *S. pyogenes* emitted significantly less bioluminescence compared to those immunized with the PBS control (Figure 7 B, Two-Way ANOVA *p*<0.05) and significantly fewer bacteria were present in the nasopharynx on the final day (Figure 7 C, Mann-Whitney Test *p*<0.05). Specific IgG reactive against heat-killed *S. pyogenes* was detected in blood samples taken a week before challenge in vaccinated mice compared with controls (Figure 7 D).

### Evaluation of the protective efficacy of SpyCEP vaccine in a nasal challenge model using BPI

**Figure 8:**
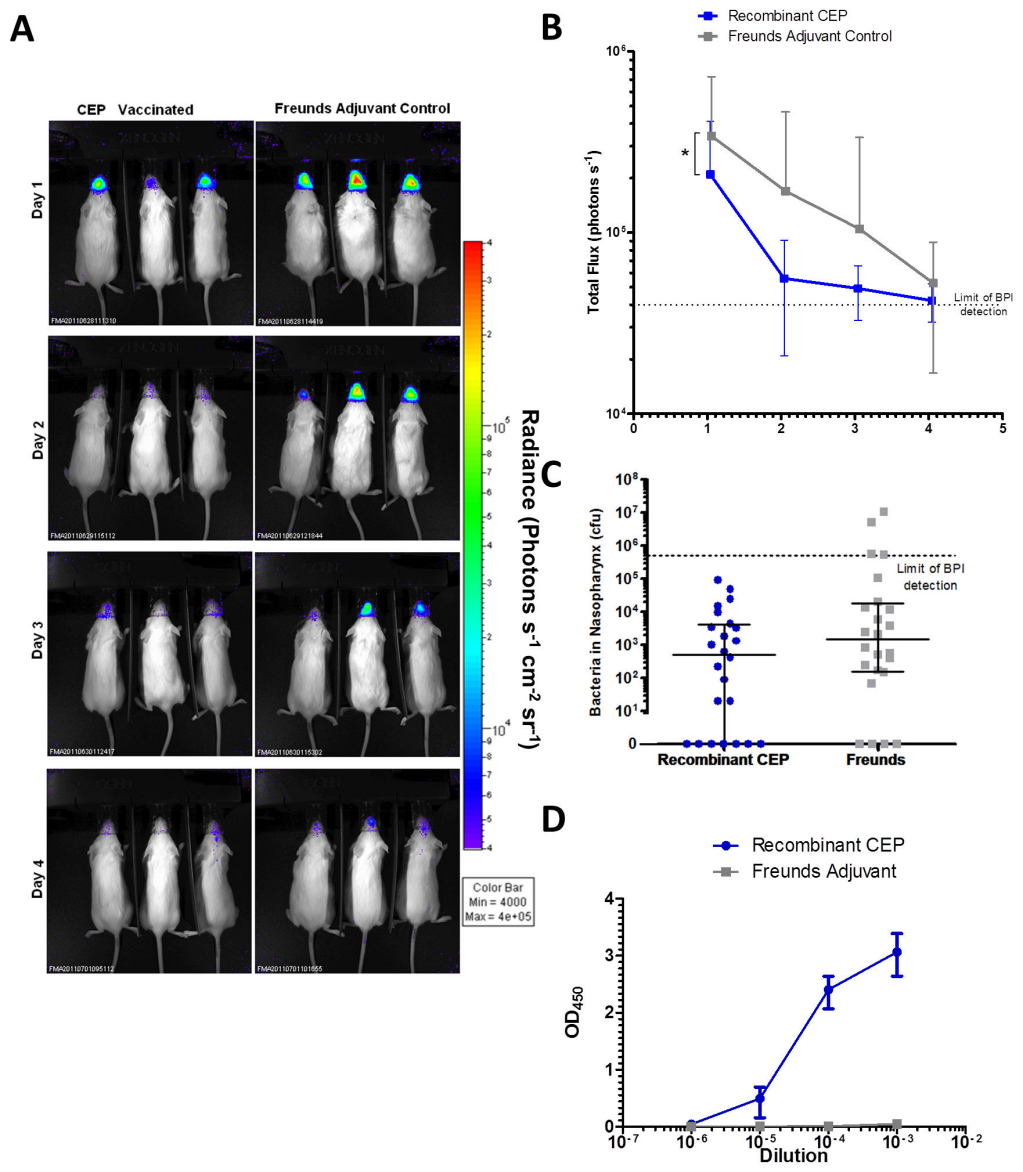
BPI to determine protective efficacy of a vaccine based on SpyCEP. Mice were immunized with either Freund’s adjuvant, or SpyCEP + Freund’s adjuvant, and then challenged with H347::lux. The mice were imaged for 4 days post infection using an IVIS 100 system (A). Total flux from mice in each group was compared over the time course (B, Repeated measures Two way ANOVA with Bonferroni correction, * *p*<0.05) Colony counts were obtained from dissections performed on the final day of infection (C). Serum antibody responses against SpyCEP from tail bleeds taken before infections were measured by ELISA (D) Error bars indicate mean and SD.

The protective efficacy of a vaccine candidate based on SpyCEP was then evaluated using the same protocol (Figure 8. A). Extrapolating from bioluminescence data alone, the SpyCEP vaccine reduced the bioluminescent signal from mice over the course of the infection compared to control mice vaccinated with Freund’s Adjuvant (Figure 8 B). However, at the final time point (Figure 8 C), there was no significant difference in bacterial numbers within the nasopharynx, when dissected. Taken together, the data show that SpyCEP vaccination was not as effective as a vaccine based on a whole cell vaccination, but do not rule out its use as a component of a multi-component vaccine. 

IgG specific against recombinant CEP were detected in blood samples taken a week before challenge in vaccinated mice compared with the Freund’s adjuvant-vaccinated controls (Figure 8 D).

## Discussion

BPI is a technique with great potential for studying infectious diseases *in vivo*. In this work, we have developed a new bioluminescent *emm*75 pharyngitis strain of *S. pyogenes* that can be used to monitor nasopharyngeal infection and have applied the model to determine the role of SpyCEP as a component of a vaccine to reduce nasopharyngeal burden.

The clinical strain of *S. pyogenes* was rendered bioluminescent through transformation with either a plasmid-based construct, or through the integration of a single copy of the *luxABCDE* operon into the chromosome. Although the multi-copy plasmid construct conferred greater brightness to *S. pyogenes* than the integrating construct, its poor stability without antibiotic selection precluded use *in vivo* where delivery of antibiotic would be problematic.

Whilst the expression of luminescence did not significantly impair the growth rate of *S. pyogenes in vitro*, survival of the bioluminescent strains was significantly impaired *in vivo* compared with wild type and control strains during nasopharyngeal infection. In pilot studies, bioluminescent *S. pyogenes* reproduced suppurative inflammation in the nasopharynx similar to that elicited by infection with wildtype *emm75 S. pyogenes* at day 3 of infection (data not shown). However inflammation completely resolved by day 7 in contrast to previous studies with the parent strain [17], consistent with reduced longevity and severity of infection. 

This reflected a general reduction in competitiveness in the nasopharynx compared to isogenic strains, which appeared to be specifically attributable to the lux operon as opposed to any disruption to the target of insertion. The reactions performed by the *luxAB* genes and the *luxCDE* genes are both energy intensive, although the latter genes theoretically account for 67% of the energy expenditure in this reaction [26]. We cannot speculate on which gene(s) of the *luxABCDE* operon, may be responsible for the observed fitness burden in *S. pyogenes* without disruption of the individual components, and testing in comparison with the bioluminescent strain. These findings corroborate previous reports, where bioluminescence has been shown to confer fitness costs in certain situations [27], and this may limit use of the *luxABCDE* operon in research studies aimed at determining subtle phenotypic differences; other bioluminescence systems may affect fitness differently .

We have demonstrated that BPI signal intensity *in vivo* is proportional to bacterial viable counts, a finding in keeping with previous studies with different bacteria [4], and providing further rationale for the use of BPI to reduce animal use by longitudinal monitoring. In this study we have measured bioluminescence from a nasopharyngeal focus of infection and found that flux from the nasopharyngeal region of interest correlated with CFU. The correlation of bioluminescent flux (photons s^-1^) with bacterial counts *in vivo* was not perfect however, and is known to be affected by a range of factors including bacterial growth phase, tissue oxygen tension, use of anaesthetic drugs, body tissue density, and fur colour [28].

BPI during intranasal infection revealed a peak in the bioluminescent signal post inoculation, which then fell below the limit of detection within the majority of mice after five days. The primary focus of infection in this model was in the ethmoturbinates, whereas a previous report found NALT to be the primary focus of infection [16]. Although *S. pyogenes* were detected in the NALT, the contrasting findings may be due to genetic and phenotypic differences between the parental bacterial strains used [29] or the strain of mouse. In the current study, an *emm*75 clinical throat isolate was used for all studies, while the commercially available bioluminescent *emm*49 isolate was derived from strain 591, a skin isolate [30].

During experiments, bioluminescent *S. pyogenes* produced an unexpected signal from the genitalia of some mice, corresponding to infection of the lower vaginal tract, suggesting direct inoculation through grooming or airborne transmission within the cage (Figure S1). This underlines the potential for bacteria used in animal experiments to be transmitted beyond the intended site of infection and demonstrates the ability of BPI to identify niches of infection that were not previously suspected.

Bioluminescent *S. pyogenes* has previously been used to determine the protective efficacy of a C5a peptidase based vaccine against NALT colonization [6]. It has also been used to evaluate the protective effect of passive immunization against J8, an M protein derivative, on intraperitoneal infection [4]. In this study, we used BPI to determine if protective efficacy of vaccines against *S. pyogenes* infection in the nasopharynx could be measured, using SpyCEP as a candidate antigen, and heat-killed *S. pyogenes* as a positive control, as a proof of principle. We observed that the heat-killed *S. pyogenes* vaccine significantly reduced streptococcal numbers in the nasopharynx, as determined by BPI throughout the experiment and also by viable counts from dissected tissues obtained on the final day of the study. BPI also demonstrated that a SpyCEP based vaccine reduced bioluminescence over the course of infection compared to Freund’s adjuvant alone. Consistent with BPI data obtained on day 4 however, bacterial counts in nasal tissues at the end of the study were not significantly different between the two groups, although by this time point, signals were close to the limit of detection. While less effective than a whole bacterial cell vaccine together with previous work [9,10,12,13], the data do not rule out use of conserved components of the cell wall such as SpyCEP as a part of a multi-component vaccine.

In summary, despite the fitness costs incurred by the expression of the *lux* operon, bioluminescent *S. pyogenes* provide a useful model for short term infection monitoring in the nasopharynx and could be used to rapidly and non-invasively screen for *S. pyogenes* vaccine efficacy.

## Supporting Information

Figure S1 **Colonisation of the Genitalia with bioluminescent *S. pyogenes* during intranasal infection.** Mice intranasally infected with *S. pyogenes* occasionally produced a bioluminescent signal from their genitalia during time course experiments.
(TIF)

## References

[1] Carapetis JR, Steer AC, Mulholland EK, Weber M (2005) The global burden of group A streptococcal diseases. Lancet Infect Dis 5: 685–694. doi:10.1016/S1473-3099(05)70267-X. PubMed: 16253886. 16253886

[2] Steer AC, Batzloff MR, Mulholland K, Carapetis JR (2009) Group A streptococcal vaccines: facts versus fantasy. Curr Opin Infect Dis 22: 544–552. doi:10.1097/QCO.0b013e328332bbfe. PubMed: 19797947. 19797947

[3] Dale JB, Penfound Ta, Chiang EY, Walton WJ (2011) New 30-valent M protein-based vaccine evokes cross-opsonic antibodies against non-vaccine serotypes of group A streptococci. Vaccine 29: 8175–8178. doi:10.1016/j.vaccine.2011.09.005. PubMed: 21920403. 21920403 PMC3195966

[4] Sheel M, Pandey M, Good MF, Batzloff MR (2010) Correlation between bioluminescence and bacterial burden in passively protected mice challenged with a recombinant bioluminescent M49 group A streptococcus Strain. Clin Vaccine Immunol 17: 127–133. doi:10.1128/CVI.00256-09. PubMed: 19889937. 19889937 PMC2812104

[5] Steer AC, Law I, Matatolu L, Beall BW, Carapetis JR (2009) Global emm type distribution of group A streptococci: systematic review and implications for vaccine development. Lancet Infect Dis 9: 611–616. doi:10.1016/S1473-3099(09)70178-1. PubMed: 19778763. 19778763

[6] Park HS, Cleary PP (2005) Active and passive intranasal immunizations with streptococcal surface protein C5a peptidase prevent infection of murine nasal mucosa-associated lymphoid tissue, a functional homologue of human tonsils. Infect Immun 73: 7878–7886. doi:10.1128/IAI.73.12.7878-7886.2005. PubMed: 16299278. 16299278 PMC1307028

[7] Schulze K, Medina E, Chhatwal GS, Guzmán CA (2003) Stimulation of long-lasting protection against *Streptococcus* *pyogenes* after intranasal vaccination with non adjuvanted fibronectin-binding domain of the SfbI protein. Vaccine 21: 1958–1964. doi:10.1016/S0264-410X(02)00803-4. PubMed: 12706684. 12706684

[8] Kawabata S, Kunitomo E, Terao Y, Nakagawa I, Kikuchi K et al. (2001) Systemic and mucosal immunizations with fibronectin-binding protein FBP54 induce protective immune responses against *Streptococcus* *pyogenes* challenge in mice. Infect Immun 69: 924–930. doi:10.1128/IAI.69.2.924-930.2001. PubMed: 11159987. 11159987 PMC97971

[9] Rodríguez-Ortega M, Norais N, Bensi G, Liberatori S, Capo S et al. (2006) Characterization and identification of vaccine candidate proteins through analysis of the group A Streptococcus surface proteome. Nat Biotechnol 24: 191–197. doi:10.1038/nbt1179. PubMed: 16415855. 16415855

[10] Bensi G, Mora M, Tuscano G, Biagini M, Chiarot E et al. (2012) Multi high-throughput approach for highly selective identification of vaccine candidates: the Group A Streptococcus case. Mol Cell Proteomics 11: M111: 015693. 22286755 10.1074/mcp.M111.015693 PMC3433891

[11] Kurupati P, Turner CE, Tziona I, Lawrenson RA, Alam FM et al. (2010) Chemokine-cleaving *Streptococcus* *pyogenes* protease SpyCEP is necessary and sufficient for bacterial dissemination within soft tissues and the respiratory tract. Mol Microbiol 76: 1387–1397. doi:10.1111/j.1365-2958.2010.07065.x. PubMed: 20158613. 20158613 PMC2904501

[12] Turner CE, Kurupati P, Wiles S, Edwards RJ, Sriskandan S (2009) Impact of immunization against SpyCEP during invasive disease with two streptococcal species: *Streptococcus* *pyogenes* and *Streptococcus* *equi* . Vaccine 27: 4923–4929. doi:10.1016/j.vaccine.2009.06.042. PubMed: 19563892. 19563892 PMC2759039

[13] Fritzer A, Senn BM, Minh DB, Hanner M, Gelbmann D et al. (2010) Novel conserved group A streptococcal proteins identified by the antigenome technology as vaccine candidates for a non-M protein-based vaccine. Infect Immun 78: 4051–4067. doi:10.1128/IAI.00295-10. PubMed: 20624906. 20624906 PMC2937439

[14] Andreu N, Zelmer A, Wiles S (2011) Noninvasive biophotonic imaging for studies of infectious disease. FEMS Microbiol Rev 35: 360–394. doi:10.1111/j.1574-6976.2010.00252.x. PubMed: 20955395. 20955395 PMC3084502

[15] Russell WMS, Burch RL (1959) The Principles of Humane Experimental Technique. Methuen.

[16] Park HS, Francis KP, Yu J, Cleary PP (2003) Membranous cells in nasal-associated lymphoid tissue: a portal of entry for the respiratory mucosal pathogen group A streptococcus. J Immunol 171: 2532–2537. PubMed: 12928403. 12928403 10.4049/jimmunol.171.5.2532

[17] Alam FM, Turner CE, Smith K, Wiles S, Sriskandan S (2013) Inactivation of the CovR/S virulence regulator impairs infection in an improved murine model of *Streptococcus* *pyogenes* naso-pharyngeal infection. PLOS ONE 8: e61655. doi:10.1371/journal.pone.0061655. PubMed: 23637876. 23637876 PMC3636223

[18] Manetti AGO, Zingaretti C, Falugi F, Capo S, Bombaci M et al. (2007) *Streptococcus* *pyogenes* pili promote pharyngeal cell adhesion and biofilm formation. Mol Microbiol 64: 968–983. doi:10.1111/j.1365-2958.2007.05704.x. PubMed: 17501921. 17501921

[19] Beard SJ, Salisbury VC, Lewis RJ, Sharpe JA, MacGowan AP (2002) Expression of *lux* genes in a clinical isolate of *Streptococcus* *pneumoniae*: using bioluminescence to monitor gemifloxacin activity. Antimicrob Agents Chemother 46: 538–542. doi:10.1128/AAC.46.2.538-542.2002. PubMed: 11796373. 11796373 PMC127039

[20] Yanisch-Perron C, Vieira J, Messing J (1985) Improved M13 phage cloning vectors and host strains: nucleotide sequences of the M13mp18 and pUC19 vectors. Gene 33: 103–119. doi:10.1016/0378-1119(85)90120-9. PubMed: 2985470. 2985470

[21] Riedel CU, Monk IR, Casey PG, Morrissey D, O’Sullivan GC et al. (2007) An improved luciferase tagging system for *Listeria* *monocytogenes* allows real-time monitoring *in* *vivo* and *in* *vitro* . Appl Environ Microbiol 73: 3091–3094. doi:10.1128/AEM.02940-06. PubMed: 17351089. 17351089 PMC1892880

[22] Sriskandan S, Unnikrishnan M, Krausz T, Cohen J (1999) Molecular analysis of the role of streptococcal pyrogenic exotoxin A ( SPEA ) in invasive soft-tissue infection resulting from *Streptococcus* *pyogenes* . Mol Microbiol 33: 778–790. doi:10.1046/j.1365-2958.1999.01525.x. PubMed: 10447887. 10447887

[23] Biswas I, Jha JK, Fromm N (2008) Shuttle expression plasmids for genetic studies in *Streptococcus* *mutans* . Microbiology 154: 2275–2282. doi:10.1099/mic.0.2008/019265-0. PubMed: 18667560. 18667560 PMC4110107

[24] Turner CE, Kurupati P, Jones MD, Edwards RJ, Sriskandan S (2009) Emerging role of the interleukin-8 cleaving enzyme SpyCEP in clinical *Streptococcus* *pyogenes* infection. J Infect Dis 200: 555–563. doi:10.1086/603541. PubMed: 19591574. 19591574 PMC2820315

[25] La Rosa SL, Diep DB, Nes IF, Brede DA (2012) Construction and application of a *luxABCDE* reporter system for real-time monitoring of *Enterococcus* *faecalis* gene expression and growth. Appl Environ Microbiol 78: 7003–7011. doi:10.1128/AEM.02018-12. PubMed: 22843522. 22843522 PMC3457518

[26] Van der Meer JR, Tropel D, Jaspers M (2004) Illuminating the detection chain of bacterial bioreporters. Environ Microbiol 6: 1005–1020. doi:10.1111/j.1462-2920.2004.00655.x. PubMed: 15344926. 15344926

[27] Bose JL, Rosenberg CS, Stabb EV (2008) Effects of *luxCDABEG* induction in *Vibrio* *fischeri*: enhancement of symbiotic colonization and conditional attenuation of growth in culture. Arch Microbiol 190: 169–183. doi:10.1007/s00203-008-0387-1. PubMed: 18521572. 18521572 PMC4264523

[28] Troy T, Jekic-McMullen D, Sambucetti L, Rice BW (2004) Quantitative comparison of the sensitivity of detection of fluorescent and bioluminescent reporters in animal models. Mol Imaging 3: 9–23. doi:10.1162/153535004773861688. PubMed: 15142408. 15142408

[29] Sumby P, Whitney AR, Graviss EA, DeLeo FR, Musser JM (2006) Genome-Wide Analysis of Group A Streptococci Reveals a Mutation That Modulates Global Phenotype and Disease Specificity. PLoS Pathog 2: 0041-0049 10.1371/journal.ppat.0020005 PMC1354197 16446783

[30] Beyer-Sehlmeyer G, Kreikemeyer B, Hörster A, Podbielski A (2005) Analysis of the growth phase-associated transcriptome of *Streptococcus* *pyogenes* . Int J Med Microbiol 295: 161–177. doi:10.1016/j.ijmm.2005.02.010. PubMed: 16044856. 16044856

