# Supplementary figures and images for: Non-Invasive Monitoring of Streptococcus pyogenes Vaccine Efficacy Using Biophotonic Imaging
Source: PLoS One. 2013 Nov 20;8(11):e82123. doi: 10.1371/journal.pone.0082123 (PMC3835743; doi:10.1371/journal.pone.0082123)

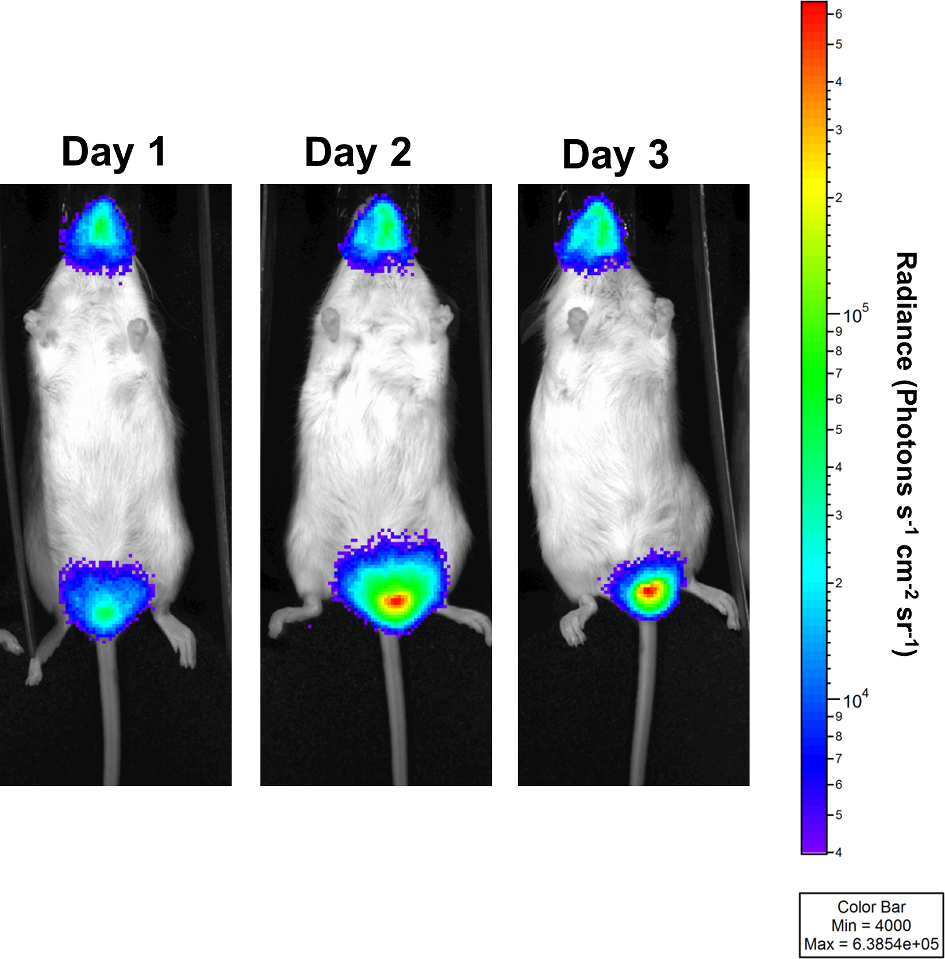

Supplement: Figure S1 — Colonisation of the Genitalia with bioluminescent S. pyogenes during intranasal infection. Mice intranasally infected with S. pyogenes occasionally produced a bioluminescent signal from their genitalia during time course experiments. (TIF) [file pone.0082123.s001.tif]
